# Regulation of cocaine seeking behavior by locus coeruleus noradrenergic activity in the ventral tegmental area is time- and contingency-dependent

**DOI:** 10.3389/fnins.2022.967969

**Published:** 2022-08-05

**Authors:** Wojciech B. Solecki, Michał Kielbinski, Michał Wilczkowski, Katarzyna Zajda, Karolina Karwowska, Bernacka Joanna, Zenon Rajfur, Ryszard Przewłocki

**Affiliations:** ^1^Department of Neurobiology and Neuropsychology, Institute of Applied Psychology, Jagiellonian University, Kraków, Poland; ^2^Department of Brain Biochemistry, Maj Institute of Pharmacology Polish Academy of Sciences, Kraków, Poland; ^3^Laboratory of Pharmacology and Brain Biostructure, Department of Pharmacology, Maj Institute of Pharmacology Polish Academy of Sciences, Kraków, Poland; ^4^Department of Biosystems Physics, Institute of Physics, Jagiellonian University, Kraków, Poland; ^5^Department of Molecular Neuropharmacology, Maj Institute of Pharmacology Polish Academy of Sciences, Kraków, Poland

**Keywords:** ventral tegmental area, noradrenaline, locus coeruleus, cocaine seeking, conditional cues

## Abstract

Substance use disorder is linked to impairments in the ventral tegmental area (VTA) dopamine (DA) reward system. Noradrenergic (NA) inputs from locus coeruleus (LC) into VTA have been shown to modulate VTA neuronal activity, and are implicated in psychostimulant effects. Phasic LC activity controls time- and context-sensitive processes: decision making, cognitive flexibility, motivation and attention. However, it is not yet known how such temporally-distinct LC activity contributes to cocaine seeking. In a previous study we demonstrated that pharmacological inhibition of NA signaling in VTA specifically attenuates cocaine-seeking. Here, we used virally-delivered opsins to target LC neurons for inhibition or excitation, delivered onto afferents in VTA of male rats seeking cocaine under extinction conditions. Optogenetic stimulation or inhibition was delivered in distinct conditions: upon active lever press, contingently with discreet cues; or non-contingently, i.e., throughout the cocaine seeking session. Non-contingent inhibition of LC noradrenergic terminals in VTA attenuated cocaine seeking under extinction conditions. In contrast, contingent inhibition increased, while contingent stimulation reduced cocaine seeking. These findings were specific for cocaine, but not natural reward (food) seeking. Our results show that NA release in VTA drives behavior depending on timing and contingency between stimuli – context, discreet conditioned cues and reinforcer availability. We show that, depending on those factors, noradrenergic signaling in VTA has opposing roles, either driving CS-induced drug seeking, or contributing to behavioral flexibility and thus extinction.

## Introduction

Substance use disorder (SUD) is a phenomenon with multiple functional axes, including increased incentive salience of drug-related cues, attenuation of normal rewards with concomitant vulnerability to stress as well as impaired impulse control, executive function and memory (Carmack et al., 2016; Koob and Volkow, 2016; Uhl et al., 2019). Dopamine (DA) neurotransmission is implicated to some degree in all of those aspects, and thus, DAergic signaling in the mesocorticolimbic system is at the center of addiction etiology (Volkow et al., 2009, 2011; Koob and Volkow, 2016). However, the search for mechanisms of addiction increasingly encompasses other neurotransmitter systems. Noradrenaline (NA), a catecholamine transmitter sharing many biochemical similarities with DA arises as an important factor, especially for stimulant drugs, which are known to affect both DA and NA release and uptake (Weinshenker and Schroeder, 2007; Schmidt and Weinshenker, 2014). The possible relevance of NA for SUD extends well beyond mediating acute stimulant effects, however. The locus coeruleus (LC) NA system is involved in motivation, salience encoding, memory processing, adaptability and flexibility of action choice and effort-versus-reward judgments (Aston-Jones and Cohen, 2005; Sara, 2009; Sara and Bouret, 2012; Kalwani et al., 2014; Carmack et al., 2016; Jahn et al., 2018; Hauser et al., 2019; Walton and Bouret, 2019; Poe et al., 2020). Of particular note is the evidence linking synchronized phasic firing of LC neurons to responses to discreet events, such as presentation of conditioned stimuli (CS) (Sara and Segal, 1991; Totah et al., 2018, 2019). The ability of NA signals to rapidly shift cognitive resources (attention, perception, focus) toward salient stimuli in a context-dependent manner is relevant for drug craving, which typically manifests in response to highly salient drug-related cues, with the context in which these cues are presented serving to modulate both the behavioral response and the patterns of neural activity that accompany it (Robinson and Berridge, 1993; Crombag et al., 2008; Jasinska et al., 2014; Zilberman et al., 2019).

Although cross-talk between DA and NA in their downstream targets in the forebrain is well-established (El Mansari et al., 2010; Xing et al., 2016; Ranjbar-Slamloo and Fazlali, 2020), comparatively little is known about potential direct regulation of the DA system by NA released from LC afferents in the ventral tegmental area (VTA). There is evidence indicating that VTA receives strong NAergic inputs (Mejias-Aponte et al., 2009; Mejias-Aponte, 2016). NA in the VTA can act dose- and context-dependently to inhibit, or, conversely, upregulate DA neuron firing both directly and indirectly, e.g., by interacting with GABAergic interneurons (Grenhoff et al., 1993; Paladini and Williams, 2004; Goertz et al., 2015; Pradel et al., 2018). Importantly, NA, *via* presynaptic receptors on glutamatergic and GABAergic terminals (Jiménez-Rivera et al., 2012; Velásquez-Martinez et al., 2012; Williams et al., 2014; Velásquez-Martínez et al., 2015) could shape the responses of VTA neurons to phasic excitatory inputs, such as pedunculopontine tegmental and laterodorsal tegmental nuclei, inhibitory inputs, such as rostromedial tegmentum (RMTg), as well as indirect inhibition from lateral habenula projections onto GABAergic neurons (Hou et al., 2002; Bourdy and Barrot, 2012; Velásquez-Martínez et al., 2015; Hu et al., 2020).

The sum total of these interactions is consistent with a facilitation of DA signaling by NA. LC activity, *via* adrenergic receptors (ARs) in the VTA, increases DA neuron firing (Grenhoff et al., 1993) and enhances phasic DA release in nucleus accumbens (Goertz et al., 2015; Park et al., 2017; Kielbinski et al., 2019), which in turn is associated with drug seeking (Phillips et al., 2003; Stuber et al., 2005). This LC-VTA circuit is susceptible to local pharmacological manipulation. Previously, we demonstrated decreased cocaine seeking under extinction conditions after α_1_-AR antagonist infusion into VTA. Conversely, infusion of the α_1_-AR agonist phenylephrine, or α_2_-AR antagonist RX821002 (which blocks autoreceptors on NAergic terminals, thus enhancing transmitter release) increased seeking (Solecki et al., 2018). In a follow-up study, we demonstrated that α_1_-AR antagonist infusion into VTA modulates CS-induced cocaine seeking in a novel context. We hypothesized that this involved modifying the salience of discrete CS associated with cocaine as cue-induced, but not stress-induced, reinstatement was also affected (Solecki W. B. et al., 2019). These effects were highly specific to cocaine, in contrast to natural rewards such as food pellets (Solecki et al., 2018; Solecki W. B. et al., 2019). Furthermore, a recent study (Deal et al., 2020) has demonstrated that alcohol drinking behavior in an operant paradigm can be bi-directionally manipulated by applying direct optogenetic stimulation to LC neurons. In that study, low-frequency (tonic) stimulation resulted in increased alcohol intake, while high-frequency (phasic) stimulation resulted in a decrease.

The present study aims to extend upon these two lines of work by determining whether cocaine seeking can be controlled by manipulating the LC-VTA circuit, and by determining the relationship between drug-associated cues and the effects of NA. We used opsins virally delivered to the LC in combination with laser stimulation delivered into the VTA to either, inhibit, or stimulate NA release from LC-VTA efferents during cocaine seeking under extinction conditions. Optogenetic stimulation or inhibition was delivered in two paradigms: *contingent* or *non-contingent*. Contingent manipulation was performed by coupling optogenetic stimulation or inhibition to active lever presses and cue presentation during cocaine or food seeking under extinction conditions. Conversely, non-contingent inhibition was delivered uniformly throughout the session, independently of lever presses. The intention was to introduce temporally selective modulation of LC inputs into the VTA during either the processing of discreet cues associated with operant responses, or more broadly during the processing of the contextual cues associated with the experimental session, respectively. Non-contingent activation of inhibitory opsins (ArchT), which presumably inhibits NA release, resulted in attenuated cocaine seeking. Contingent manipulation elicited distinctly bi-directional effects: activation of ArchT (presumably leading to inhibition of NA release) increased cocaine seeking behavior, while activation of ChR2 (presumably leading to increased NA release) – attenuated it. Together, these findings are consistent with the notion that NAergic activity, in response to both contextual and discreet cues, is capable of time-dependent modulation of drug seeking responses by acting at the level of DA neurons in VTA.

## Materials and methods

### Subjects

Male Sprague Dawley Tyrosine hydroxylase (TH) IRES-Cre rats^+/–^ transgenic rats, SD-Th-cre ^tm1sage^ (Liu et al., 2016) were bred in the Institute of Zoology and Biomedical Research, Jagiellonian University (Krakow, Poland) under a breeding license with Horizon Discovery (Vienna, Austria). Experiments were performed during the light phase of the light/dark cycle. Experiments were conducted according to the EU Guide for the Care and Use of Laboratory Animals and were approved by the Committee on the Ethics of Animal Experiments at the Jagiellonian University.

### Virus transduction

Cre-dependent adeno-associated viruses (AAVs) inducing archaeorhodopsin (Arch 3.0) along with EYFP (AAV5-EF1a-DIO-eArchT3.0-EYFP) or channelrhodopsin (ChR2) along with EYFP (rAAV5-EF1a-DIO-hchR2-(H134R)-EYFP) as well as control Cre-dependent AAVs expressing EYFP only (AAV5-EF1a-DIO-EYFP) were obtained from the University of North Carolina Viral Core. Details of AAV vectors and their micro-infusion are presented in the supplement.

### Immunohistochemistry

After perfusion and decapitation, brains were sectioned with a vibratome (model VT1200, Leica Biosystems, Germany) into 50 μm coronal slices for further immunohistological procedures. The supplement presents the details of the tyrosine hydroxylase (TH) and enhanced yellow fluorescent protein (EYFP) staining. The efficiency and specificity of viral transductions were evaluated by confocal microscopy (LSM 710 on Axio Observer Z1 microscope; Zeiss, EC Plan-Neofluar 10x/0.30 M27 objective), with ImageJ software (Schneider et al., 2012) used for image analysis. Co-localization between EYFP and TH immunoreactivity was assessed, as described in the supplement.

### Intra-ventral tegmental area (VTA) optical fiber implantation

Four weeks after virus microinfusion surgery, all rats for behavioral experiments underwent optical fiber implantation procedure, as described previously (Solecki W. et al., 2019). Dual (bilateral) fiber-optic cannulas (DFC_200/245-0.37_DF1.0_FLT, Doric Lenses Inc., Quebec, Canada) were placed dorsal to the anterior part of VTA (AP –5.2 mm, ML ± 0.5 mm, DV –7.4 to 7.8 mm from the Bregma). Next, four anchor screws (Agnthos, Sweden) were mounted in the skull and dental cement (Duracryl, SpofaDental, Czech Republic) was used to ensure stability of the optical fiber. After surgery each subject underwent post-surgery care detailed in the supplement.

### Cocaine seeking under extinction conditions

SD-TH-Cre^+^ rats were implanted with I.V. catheter and after recovery period and food-self-administration pre-training underwent cocaine self-administration training (Solecki W. et al., 2019) (Figure 2A; details in the supplement). Briefly, rats were trained in standard operant chambers (Med Associates, St. Albans, VT, United States) under a fixed ratio 1 (FR1) schedule of reinforcement during which lever press led to an intravenous cocaine infusion (0.18 mg over 6 s, ∼0.5 mg/kg) and the conditional stimulus (CS) presentation (tone + stimulus light for 6 s). Each active lever press was followed by a 20 s timeout during which lever pressing had no programed consequences. Similarly, inactive lever presses had no programed consequences. Each rat received 2-h daily training sessions for 9-10 consecutive days. During last three self-administration sessions habituation to optic-fiber was introduced to minimize potential artifacts during tests using optogenetic modulation. Immediately after the end of the training session each rat was connected to optic-fiber and remained in the skinner box (house light on, no levers available) for 15 min.

Next, all rats underwent 3 days of forced abstinence in their home cages. On withdrawal day 3 (WD 3) cocaine-seeking under extinction conditions combined with optogenetic modulation of the VTA was performed as previously described (Solecki W. et al., 2019). In this test, rats engage in previously conditioned instrumental responding (i.e., lever presses) which leads to no cocaine infusions; with a number of active lever presses serving as a measure of cocaine seeking and craving. Immediately prior to test, each subject’s bilateral intra-VTA optic-fiber was connected with the laser source, immediately prior to the cocaine-seeking test. Next, animals were placed in operant chambers for 30 min during which optogenetic modulation was applied. During this period, active lever presses led to the CS presentation alone with no cocaine delivery, whereas pressing the inactive lever had no programed consequences. Inhibition of the noradrenergic VTA afferents from LC expressing Arch3.0 was applied either non-contingently (1 pulse, 6 s every 12 s, 10 mW at the optical fiber tip) or contingently upon active lever press (1 pulse for 6 s, 10 mW at the optical fiber tip). In contrast, stimulation of LC TH^+^ efferents expressing ChR2 was applied only contingently upon active lever press (360 × 10 ms pulses at 60 Hz during CS presentation i.e., 6 s duration).

### Food self-administration and food seeking under extinction conditions

Following 6 days of recovery from intra-VTA cannula implantation surgery, rats underwent food self-administration training, as published previously (Solecki W. et al., 2019) (Figure 3A). Briefly, animals were restricted to 90% of their free-feeding levels for 2–3 days. One day prior to training, 20–30 food pellets (Dustless Precision Pellets F0021; BioServ, NJ, United States) were placed into the home cage to introduce the rats to the new food. Rats were trained in the same operant chambers used in the cocaine self-administration experiment, as described above (Med Associates, St. Albans, VT, United States). Each rat was placed in the chamber with a fixed ratio 1 (FR1) schedule of sucrose reinforcement, where an active lever press led to the delivery of a 45-mg food pellet and the simultaneous presentation of a 6 s audio-visual cue (tone + cue light presentation), followed by a 10 s timeout during which time the lever was retracted. Inactive lever presses had no programed consequence. Rats received 1 h training sessions over 9 consecutive days and then underwent a three-day period of forced abstinence, during which they had no exposure to food pellets, the operant chamber, or food-associated contextual or discrete cues.

On withdrawal day 3 (WD 3), food-seeking under extinction conditions was performed, during which every active lever press led to the CS presentation but no food delivery. Inactive lever presses had no programed consequences. Optogenetic stimulation of VTA activity delivered contingently was performed as described above.

### Locomotor activity

48 h after WD 3 testing all rats underwent open field test to evaluate effects of optogenetic stimulation or inhibition on locomotor activity and anxiety-like behaviors. The center of a square apparatus (80 × 80 × 60 cm with matte black walls and floor) was illuminated with a light intensity of 5 20 lux (5 lux on periphery and 20 lux in the center). Immediately after the intra-VTA optical fibers were connected to the laser source, animals were placed in the center of the apparatus and left inside for 15 min during which stimulation or inhibition was applied as described above. Each rat behavior was recorded and analyzed using ANY-maze video-tracking software (Stoelting Europe, Ireland). Distance traveled during the test was used as a measure of locomotor activity, whereas time spent in the center zone of the apparatus (circular zone in the middle of the apparatus, with 13 cm radius and 20 lux illumination) was used as an index of anxiety-like (neophobia) behaviors. The apparatus was cleaned using 70% ethanol and dried with a cleaning cloth between each rats.

### Real-time dynamic place preference

The place preference testing was performed 24 h after the open field test in real-time dynamic conditioned place preference/aversion paradigm (Tan et al., 2012), based on our protocol published previously (Solecki W. et al., 2019) (Figure 4A). The testing was performed in a custom-made apparatus (black Plexiglas 80 × 80-cm arena with 60-cm-high black walls) consisting of two conditioning chambers (27 × 27 × 40 cm) separated by a central platform (15 × 7 × 40 cm). The two conditioning chambers differed in their visual and tactile cues (described in detail in the supplement). The real time conditioned place preference (RT-CPP) consisted of three stages: a preconditioning test (day 1), conditioning (days 2 and 3), and a postconditioning test (24 h after last conditioning). During the preconditioning and postconditioning tests, rats were placed individually on the central platform of the apparatus with free access to both chambers for 15 min. After the preconditioning phase, one chamber was paired with laser stimulation (laser-paired chamber) and the other with no stimulation (control chamber). The assignment of treatments (laser-paired vs. control) to the chambers was counterbalanced (each chamber served as laser-paired with equal frequency). During conditioning, the rats were connected to a laser source (473 nm for optogenetic stimulation and 561 nm for inhibition), immediately before being placed in the apparatus. Power density at the fiber tip was set under 10 mW/mm^2^. Importantly, for consistency across RT-CPP stages, each subject was connected to a laser source before pre- and postconditioning, however no laser modulation was applied. Next, animals were placed on the central platform and had free access to both chambers for 30 min during which each entrance to the laser-paired chamber triggered laser stimulation (473 nm light pulses at 60 Hz for 6 s at 12 s interval with 1 pulse lasting 10 ms) *via* ANY-maze software combined with pulse generator (OTPG_4, Doric Lenses Inc., Quebec, Canada). Each exit from the laser-paired chamber terminated ongoing laser stimulation. Entrance or exit to and from the control chamber had no consequences. All experiments were recorded by a video camera, and time spent in each chamber as well as distance traveled was measured using ANY-maze software. The RT-CPP was evaluated according to an unbiased procedure using unbiased conditioning apparatus chambers (on average, rats spent equal time in both chambers during preconditioning) and unbiased treatment assignment. Animals that displayed high preference (up to 200 s) for one of the chambers in the preconditioning phase were excluded from subsequent testing (*n* = 10). CPP score was defined as the difference in time spent in the laser-paired and control arms during the day of testing.

### Histological verification of optical fiber placement

Verification of fiber placement in the VTA was performed as described previously (Solecki W. et al., 2019). Animals were anesthetized with pentobarbital (150 mg/kg i.p., Biowet-Pulawy, Poland), and perfused with 4% paraformaldehyde. Immediately after perfusion, animals were decapitated; their brains were removed and placed in 4% paraformaldehyde for 72 h. Brains were sliced (100 μm) with the vibratome (model VT1000S, Leica Biosystems, Germany) and LC transduction and intra-VTA optic-fiber tracts were analyzed with use of fluorescent microscopy (Zeiss Axioskop 50, Germany). All data from subjects with no transduction (and thus no Arch3.0 or ChR2 expression) were included in the EYFP-expressing controls. All data from subjects with misplaced optic fiber were excluded from further analysis (Supplementary Table 1). Representative optical fibers placements are shown in Supplementary Figure 1.

### Data analysis

Behavioral effects of optogenetic stimulation and inhibition were analyzed using Student’s *t*-test, a one-way ANOVA, a two-way repeated-measures ANOVA or a three-way repeated-measures ANOVA (GraphPad Software, San Diego, CA, United States, Statistica 12.5, Stat-Soft, Poland; detailed description in the supplement), similarly to our previous studies (Solecki et al., 2018; Solecki W. et al., 2019). If there was a significant main effect or a significant interaction, a subsequent Tukey *post hoc* analysis was performed. Supplementary Table 2 presents the factors and levels of ANOVA according to the stimulation paradigm and performed behavioral tests. Statistical significance was set at *P* < 0.05. All results values are presented as the means + SEM.

## Results

### Efficacy of viral transduction

**FIGURE 1:**
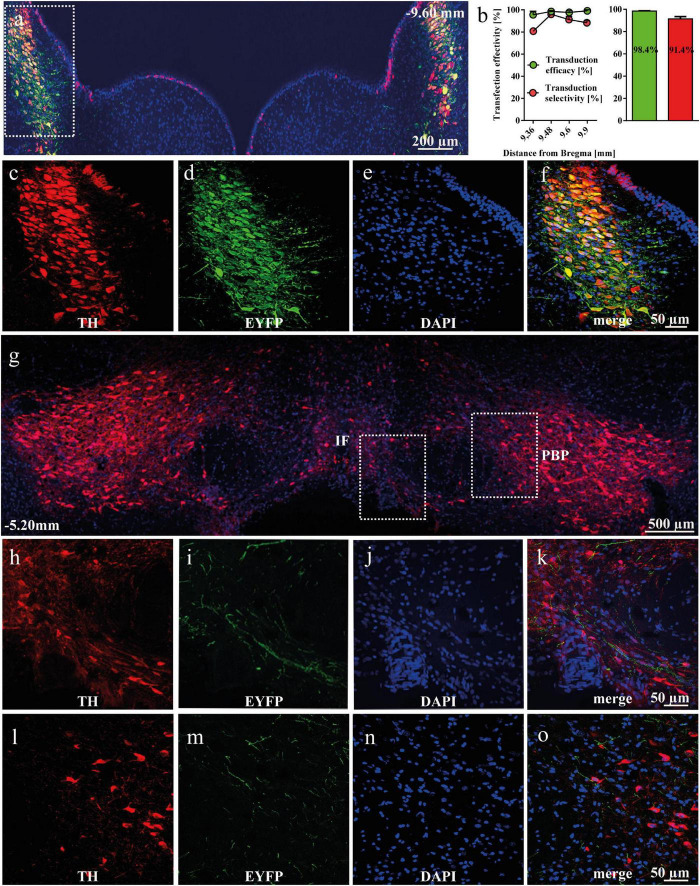
Viral transduction of noradrenergic (NA) neurons in the locus coeruleus (LC) of tyrosine hydroxylase (TH)-Cre^+^ rats. **(a)** Representative image of enhanced yellow fluorescent protein (EYFP)-expressing and TH-immunopositive cells in the LC. **(b)** The effectivity and selectivity of transduction were evaluated in TH-Cre^+^ rats (*n* = 3) 5 weeks after AAV5-EF1a-DIO-EYFP intra-LC micro-injection. Data is shown as mean ± SEM. **(c–f)** Magnified cells with TH (red), EYFP (green), DAPI (blue) and merged channels, showing co-localized TH and EYFP expression. **(g)** EYFP-expressing NA-ergic terminals from the LC are present in the ventral tegmental area (VTA). Representative panoramic image of the VTA of a rat transduced with AAV5-EF1a-DIO-EYFP into the LC. **(h–k)** Representative image of EYFP-positive terminals from the LC arriving into the IF, with TH in red, EYFP in green, DAPI in blue and merged channels. **(l–o)** Representative image of EYFP-positive terminals from the LC arriving into the PBP, with TH in red, EYFP in green, DAPI in blue and merged channels. IF, intrafascicular nucleus; PBP, parabrachial pigmentated nucleus.

After intra-LC AAV delivery, 98.4% of TH^+^ neurons in LC were expressing EYFP, indicating high efficiency of AAV transduction in the transgenic TH-Cre^+^ rats. Similarly, 91.4% of all EYFP^+^ cells were TH^+^, demonstrating high selectivity of transduction (Figures 1a–f). EYFP-positive NA-ergic projections arising from LC were detected in the anterior VTA, both in the intrafascicular (IF; Figures 1g–k) and parabrachial pigmented (PBP, Figures 1g,l–o) nuclei. A more detailed description is presented in the supplement.

**FIGURE 2:**
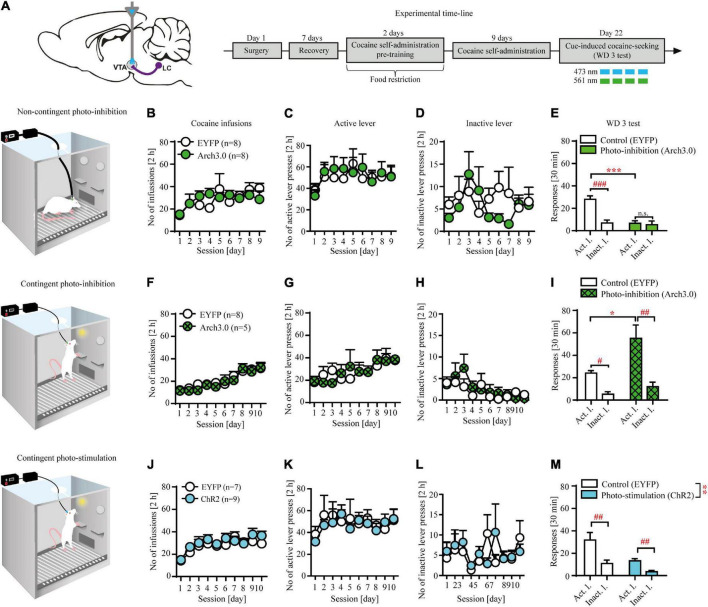
Non-contingent and contingent photo-inhibition and contingent photo-stimulation of locus coeruleus (LC) efferents in ventral tegmental area (VTA) modulates cocaine seeking under extinction conditions. **(A)** The scheme of the photo-modulation of the VTA afferents from the LC and the experimental time-line: rats, after intra-LC viral microinjections, intra-VTA optical fiber and intravenous (i.v.) catheter implantations, recovery and operant responding pre-training, were trained to self-administer cocaine (∼ 0.5 g/kg/inf) for 9-10 days (2 h/day), after which they underwent forced abstinence for 3 days. On withdrawal day 3 (WD 3), rats were tested for cocaine seeking with brief, non-contingent photo-inhibition (561 nm, 1 pulse, 6 s every 12 s) or contingent photo-inhibition (561 nm, 1 pulse for 6 s) or photo-stimulation (473 nm, 360 × 10 ms pulses at 60 Hz) where active lever presses led to the presentation of CS without cocaine delivery. **(B)** There were no differences in the number of cocaine infusions or **(C)**, **(D)** lever responses during cocaine self-administration in future non-contingent photo-inhibition groups. **(E)** Non-contingent photo-inhibition of LC noradrenergic efferents in VTA on WD 3 decreased active, but not inactive lever responding in Arch 3.0-expressing group in comparison to the control group (treatment x lever: F_(1, 28)_ = 12.44, *p* < 0.01; followed by *post hoc* test *p* < 0.001) and prevented discrimination between levers compared to the control group. **(F)** There were no differences in the number of cocaine infusions or **(G)**, **(H)** lever responses during cocaine self-administration in future contingent photo-inhibition groups. **(I)** Contingent photo-inhibition of LC noradrenergic efferents in VTA on WD 3 increased active, but not inactive lever responses in Arch 3.0-expressing group compared to the control group (treatment × lever interaction: F_(1, 22)_ = 5.51, *p* < 0.05; followed by *post hoc* test *p* < 0.05). **(J)** There were no differences in the number of cocaine infusions or **(K)**, **(L)** lever responses during cocaine self-administration in future contingent photo-stimulation groups. **(M)** Contingent photo-stimulation of LC noradrenergic efferents in VTA decreased active and inactive lever responses in ChR2-expressing group in comparison to the control group (treatment: F_(1, 28)_ = 13.37, *p* < 0.01; followed by *post hoc* test *p* < 0.001; lever interaction: F_(1, 28)_ = 19.09, *p* < 0.001; followed by *post hoc* test *p* < 0.01; treatment × lever: n.s). Data are presented as the mean ± SEM. Act. l.: active lever; Inact. l.: inactive lever. ***p < 0.001, **p < 0.01, *p < 0.05; ^###^*p* < 0.001, ^##^*p* < 0.01, ^#^*p* < 0.05; n.s. not significant.

**FIGURE 3:**
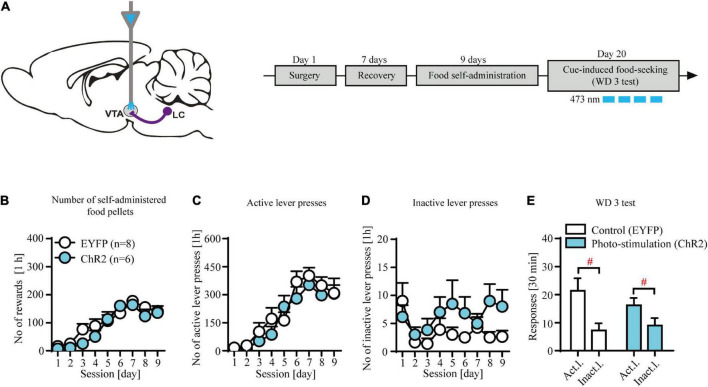
Contingent photo-stimulation of locus coeruleus (LC) noradrenergic efferents in ventral tegmental area (VTA) has no effects on food-seeking. **(A)** The scheme of the photo-modulation of the VTA afferents from the LC and the experimental time-line: rats, after intra-LC viral microinjections and intra-VTA optical fiber implantations and recovery were trained to self-administer food pellet (45-mg) for 9 days (1 h/day), after which they underwent forced abstinence for 3 days. On withdrawal 3 (WD 3), rats were tested for food seeking with brief, contingent photo-stimulation (473 nm, 360 × 10 ms pulses at 60 Hz) where active lever presses led to the presentation of CS without food delivery. **(B)** There were no differences in the number of self-administered food pellets or **(C)**, **(D)** lever responses during food self-administration in future contingent photo-stimulation groups. **(E)** Contingent photo-stimulation of LC noradrenergic efferents in VTA did not affect performance during WD 3 test, as both ChR2-expressing group and control group did not differ in levers responding (lever F_(1, 24)_ = 14.7, *p* < 0.001; followed by *post hoc* test *p* < 0.01; treatment × lever: n.s.; treatment: n.s.). Data are presented as the mean ± SEM. Act. l.: active lever; Inact. l.: inactive lever. ^#^*p* < 0.05; n.s. not significant.

**FIGURE 4:**
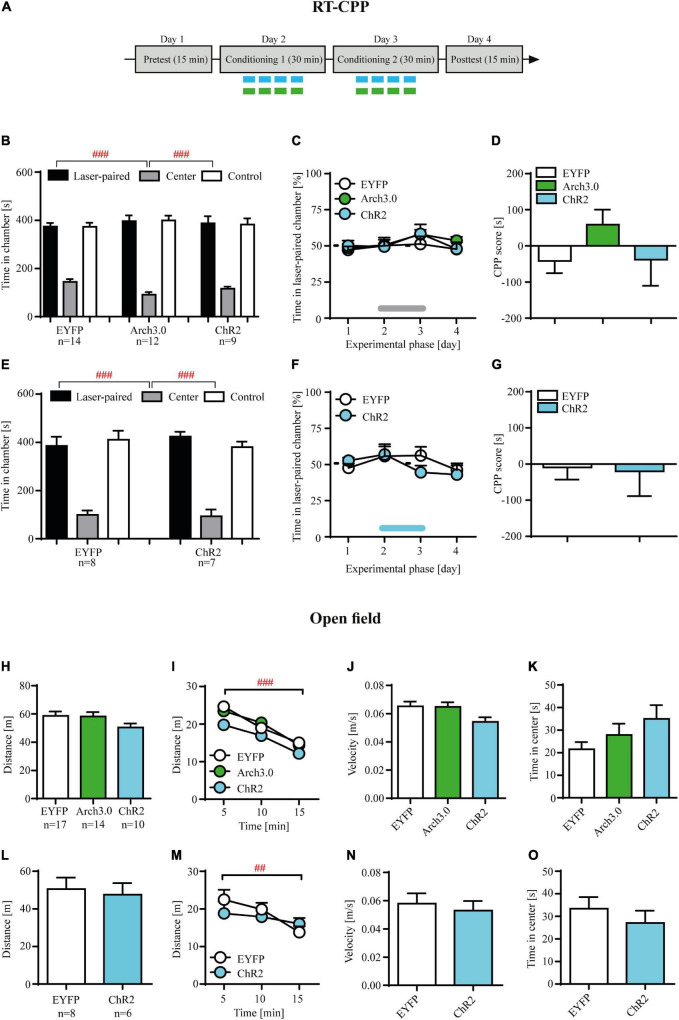
Brief photo-inhibition or photo-stimulation of locus coeruleus (LC) noradrenergic efferents in ventral tegmental area (VTA) has no effects on hedonic state measured in the real-time place preference and has no effects on locomotor activity or anxiety-like behaviors. **(A)** The experimental time-line: the real time conditioned place preference (RT-CPP) consisted of the pretekst (day 1), conditioning (days 2-3) and posttest (day 4). During the pretest and posttest rats had free access to both chambers of apparatus for 15 min, without photo-stimulation. During the conditioning one chamber was paired with laser photo-stimulation (473 nm, 10 ms light pulses at 60 Hz for 6 s at 12 s interval) or laser photo-inhibition (561 nm, 6 s at 12 s interval) and the other with no stimulation (control chamber). **(B)** There were no initial differences in time spent in conditioning chambers during pretest in control, photo-inhibited (Arch 3.0) and photo-stimulated (ChR2) cocaine-abstinent rats (chamber: F_(2, 96)_ = 209.7, *p* = 0.001; followed by *post hoc* test *p* < 0.01; treatment: n.s.; treatment × chamber: n.s.). **(C)** Photo-stimulation and photo-inhibition of LC noradrenergic efferents in VTA in cocaine-abstinent rats did not induce place preference or aversion as shown by the analysis of% of time spent in the laser-paired chamber. **(D)** These photo-modulation were insufficient in supporting the acquisition of conditioned place preference/aversion measured during posttest as shown by CPP score measure. **(E)** There were no initial differences in time spent in conditioning chambers during pretest in control and photo-stimulated cocaine-naïve rats (chamber: F_(2, 39)_ = 72.55, *p* = 0.0001; followed by *post hoc* test *p* < 0.01; treatment: n.s.; treatment × chamber: n.s.). **(F)** Photo-stimulation of LC noradrenergic efferents in VTA in cocaine- naïve rats did not induce place preference or aversion as shown by the analysis of% of time spent in the laser-paired chamber. **(G)** These photo-modulation were insufficient in supporting the acquisition of conditioned place preference/aversion measured during posttest as shown by CPP score measure. **(H)** Photo-stimulation and photo-inhibition of LC noradrenergic efferents in VTA in cocaine-abstinent rats, similar to control group did not modulate distance travelled, **(I)** habituated to the novel arena (measured as distance traveled across time), **(J)** average speed or **(K)** anxiety-like behaviors (measured as time spent in the center zone of the open field). **(L)** Photo-stimulation of LC noradrenergic efferents in VTA in cocaine-naïve rats, similar to control group did not modulate distance travelled, **(M)** distance traveled across time, **(N)** average speed or **(O)** time spent in the center zone. The line on panel C and F represents laser stimulation during conditioning. Data are presented as the mean ± SEM. ^###^*p* < 0.0001; ^##^ *p* < 0.01.

### Non-contingent optogenetic inhibition of locus coeruleus (LC) noradrenergic efferents in ventral tegmental area (VTA) attenuates cocaine seeking

Non-contingent inhibition of LC TH^+^ axon fibers in VTA reduced cocaine seeking as evidenced by decreased active but not inactive lever responding in the Arch3.0-expressing group compared to the control group (Figure 2E: treatment × lever interaction: F_(1, 28)_ = 12.44, *p* < 0.01 followed by *post hoc* test *p* < 0.001) as well as no discrimination between active and inactive lever responses (*p* > 0.05). Further examination of 5-min epochs during the 30 min session confirmed effects of non-contingent inhibition on active lever responding (Supplementary Figure 2A). The observed effects were not due to previous differences during training, as subjects from the future control (EYFP-expressing) and future inhibited (Arch3.0-expressing) groups displayed similar cocaine intake (Figure 2B: treatment × day F_(8, 112)_ = 1.06; *p* = 0.39; treatment F_(1, 14)_ = 0.02; *p* = 0.91; day F_(8, 112)_ = 2.91; *p* < 0.001) and showed no differences during cocaine self-administration training (Figures 2C,D: n.s.; Supplementary Table 2).

### Contingent optogenetic inhibition of locus coeruleus (LC) noradrenergic efferents in ventral tegmental area (VTA) facilitates cocaine seeking

Inhibition of LC TH^+^ axon fibers in VTA, contingent upon active lever press, facilitated cocaine seeking as evidenced by increased active but not inactive lever responding in the Arch3.0-expressing group compared to the control group (Figure 2J: treatment × lever interaction: F_(1, 22)_ = 5.51, *p* < 0.05 followed by *post hoc* test *p* < 0.05; for examination of 5-min epochs see Supplementary Figure 2B). The observed effects were not due to previous differences during training, as subjects from the future control (EYFP-expressing) and future inhibited groups performed similarly (cocaine intake: Figure 2F: treatment × day F_(9, 99)_ = 0.41; *p* = 0.93; treatment F_(1, 11)_ = 0.25; *p* = 0.62; day F_(9, 99)_ = 13.65; *p* < 0.001; lever responding: Figures 2G,H: n.s.; Supplementary Table 2).

### Contingent optogenetic stimulation of locus coeruleus (LC) noradrenergic efferents in ventral tegmental area (VTA) reduces cocaine seeking

Contingent optogenetic stimulation protocol attenuated cocaine seeking as evidenced by decreased active and inactive lever responding in the ChR2-expressing group compared to the control group (Figure 2M: treatment: F_(1, 28)_ = 13.37, *p* < 0.01 followed by *post hoc* test *p* < 0.001; lever interaction: F_(1, 28)_ = 19.09, *p* < 0.001 followed by *post hoc* test *p* < 0.01; treatment × lever interaction: F_(1, 28)_ = 2.53, *p* = 0.12; for examination of 5-min epochs see Supplementary Figure 2C). There were no inter-group differences during cocaine self-administration that could impact data interpretation (cocaine intake: Figure 2J: treatment × day F_(9, 126)_ = 0.61; *p* = 0.78; treatment F_(1, 14)_ = 2.62; *p* = 0.13; day F_(9, 126)_ = 5.09; *p* < 0.001; lever responding: Figures 2K,L: n.s.; Supplementary Table 2).

### Contingent optogenetic stimulation of locus coeruleus (LC) noradrenergic efferents in ventral tegmental area (VTA) has no effects on food-seeking

We observed no effects of contingent optogenetic stimulation on food seeking as evidenced by similar levels of lever responding in experimental and control groups (Figure 3E: treatment × lever interaction: F_(1, 24)_ = 0.45, *p* = 0.51; treatment F_(1, 24)_ = 0.18, *p* = 0.67; lever F_(1, 24)_ = 14.7, *p* < 0.001 followed by *post hoc* test *p* < 0.01). Likewise, subjects from the future control (EYFP-expressing) and future stimulation (ChR2-expressing) treatment groups displayed similar food intake (Figure 3B; treatment × day interaction: F_(8, 104)_ = 0.96, *p* = 0.46; treatment F_(1, 13)_ = 1.78, *p* = 0.21; day F_(8, 104)_ = 25.19, *p* < 0.001) and showed no differences during food self-administration training (Figures 3C,D; n.s.; Supplementary Table 2). Together, these results demonstrate that phasic-like optogenetic stimulation of the NA-ergic axon terminals in VTA had no effects on food seeking.

### Brief optogenetic inhibition or stimulation of locus coeruleus (LC) noradrenergic axon fibers in ventral tegmental area (VTA) has no effects on hedonic state measured in the real-time place preference

Our results demonstrating that direct (and contingent upon active lever press) optogenetic inhibition facilitates, whereas optogenetic stimulation attenuates cocaine-seeking behavior during abstinence may suggest that such optogenetic modulation may have subjective effects of its own, thus impacting cocaine-seeking indirectly. In this scenario, optogenetic inhibition of LC NA-ergic activity in VTA could have reinforcing effects and thus facilitation of cocaine seeking would be an artifact of positive reinforcement of active lever pressing. Accordingly, reduction of cocaine craving during contingent optogenetic stimulation could suggest induction of dysphoric states in cocaine-abstinent subjects, significantly declining probability of active lever pressing.

Optogenetic inhibition or stimulation of the intra-VTA NA-ergic activity in cocaine-abstinent rats had no effects on% time spent in the laser-paired chamber in the RT-CPP paradigm (Figure 4C; treatment × RT-CPP phase interaction: F_(6, 96)_ = 0.41, *p* = 0.87; treatment F_(2, 32)_ = 0.49, *p* = 0.61; RT-CPP phase F_(3, 96)_ = 2.31, *p* = 0.08). These protocols of brief intra-VTA optogenetic modulation were insufficient in supporting the acquisition of conditioned place preference/aversion measured during posttest (Figure 4D: F_(2, 32)_ = 1.52, *p* = 0.23). Finally, such results were not due to preexisting differences between control (EYFP-expressing), Arch3.0-expressing and ChR2-expressing rats, as these groups showed no differences in time spent in the control vs. laser-paired chambers during the pretest (Figure 4B; treatment: F_(2, 96)_ = 0.01, *p* = 0.99; chamber: F_(2, 96)_ = 209.7, *p* = 0.001 followed by *post hoc* test *p* < 0.01; treatment × chamber interaction: F_(4, 96)_ = 1.70, *p* = 0.15).

Similarly, optogenetic stimulation of LC TH^+^ axon fibers in VTA in cocaine-naïve rats had no effects on% time spent in the laser-paired chamber across RT-CPP conditioning phases (Figure 4F; treatment × RT-CPP phase interaction: F_(3, 39)_ = 1.45, *p* = 0.24; treatment F_(1, 13)_ = 0.14, *p* = 0.71; RT-CPP phase F_(3, 39)_ = 2.51, *p* = 0.07) nor supported acquisition of conditioned behaviors during postconditioning (Figure 4G: t = 0.15, df = 13, *p* = 0.88). Furthermore, there were no differences in the time spent in the two conditioning chambers between EYFP-expressing and ChR2-expressing rats (Figure 4E; treatment: F_(1, 39)_ = 0.0001, *p* = 0.98; chamber: F_(2, 39)_ = 72.55, *p* = 0.0001 followed by *post hoc* test *p* < 0.01; treatment × chamber interaction: F_(2, 39)_ = 0.73, *p* = 0.48).

### Brief optogenetic inhibition or stimulation of locus coeruleus (LC) noradrenergic efferents in ventral tegmental area (VTA) has no effects on locomotor activity or anxiety-like behaviors

Optogenetic inhibition or stimulation of LC efferents in the VTA in cocaine-abstinent subjects had no effects on locomotion measured as total distance traveled during 15 min open field test (Figure 4H; F_(2, 36)_ = 1.74, *p* = 0.19). In addition, there were no differences in distance traveled when analyzed across time (Figure 4I: treatment × time interaction: F_(4, 74)_ = 0.67, *p* = 0.60; treatment F_(2, 74)_ = 2.65, *p* = 0.08) and all subjects habituated to the novel arena as evidenced by decreased locomotor activity over time (time F_(2, 74)_ = 47.75, *p* < 0.001 followed by *post hoc* test *p* < 0.01). Finally, intra-VTA optogenetic modulation did not alter velocity (Figure 4J; F_(2, 36)_ = 2.75, *p* = 0.08) nor time spent in the center of the open field (Figure 4K; F_(2, 36)_ = 2.07, *p* = 0.14). Similarly, optogenetic stimulation of LC TH^+^ axon fibers in VTA in cocaine-naïve rats had no effects on locomotion nor on the pattern of open field exploration (Figures 4L–O; total distance: *t* = 0.34, df = 12, *p* = 0.74; distance across time: treatment × time interaction: F_(2, 24)_ = 2.08, *p* = 0.14; treatment F_(2, 12)_ = 0.19, *p* = 0.66; time F_(2, 24)_ = 7.84, *p* < 0.01 followed by *post hoc* test *p* < 0.01; velocity: *t* = 0.47, df = 12, *p* = 0.64; time in center: *t* = 0.84, df = 12, *p* = 0.42).

## Discussion

We previously hypothesized that AR antagonists infused into the VTA attenuate drug seeking through decreased salience of the previously entrained CS or the inhibition of signaling related to the context normally serving as “occasion setter” for drug seeking (Crombag et al., 2008; Tiffany and Wray, 2012; Sayette, 2016). Here, by contrasting contingent and non-contingent optogenetic manipulation of LC-NA terminals in the VTA, we were able to narrow down these hypotheses.

Non-contingent inhibition of archaerhodopsin-expressing LC terminals in VTA resulted in attenuated cocaine seeking, consistent with our previous studies with intra-VTA microinfusions of α-AR antagonists (Solecki et al., 2018; Solecki W. B. et al., 2019). Confounding effects can likely be excluded, as we did not observe changes in locomotor activity, dysphoria or overt signs of anxiety in the open field. Attenuation of NAergic signaling had similar effects to VTA inhibition in general: we had previously observed that non-contingent inhibition of VTA DA neurons reduces cocaine seeking (Solecki W. et al., 2019). When optogenetic inhibition was applied contingently, immediately after active lever presses, cocaine seeking was markedly increased, while *stimulation* of channelrhodopsin-expressing LC terminals upon active lever press resulted in decreased drug seeking.

In contrast to the decrease in operant responding resulting from non-contingent inhibition, which was associated with loss of discrimination between active and inactive levers, contingent stimulation induced a reduction in drug seeking while lever discrimination was maintained. Together, this suggests enhanced within-session extinction of previously learned operant behavior after LC terminal stimulation. Alternative explanations, where LC optogenetic manipulation would lead to sedation or negative reinforcement “punishing” active lever presses are, again, made less likely by negative results in open field and RT-CPP tests. This is in agreement with previous optogenetic experiments demonstrating anxiogenic effects evoked by elevated tonic (5 Hz), but not phasic (> 10 Hz), LC NA neuron firing patterns (McCall et al., 2015).

Optogenetic manipulation of the LC-VTA circuit is also similar to previously reported effects of drug infusions into VTA (Solecki et al., 2018; Solecki W. B. et al., 2019) in another aspect, namely, that it appeared to specifically alter cocaine seeking and had no effect on operant behavior associated with a natural reward, food. We previously speculated that this is due to higher salience of cocaine-paired CS versus food CS at WD 3. From a mechanistic standpoint, this could be due to plasticity in VTA DAergic neurons and concomitant alterations in DA release and reuptake dynamics, which have previously been shown to result from prolonged exposure to cocaine and/or cocaine withdrawal (Willuhn et al., 2014; Saddoris et al., 2016; Siciliano et al., 2019); these findings correspond to symptoms of SUD in humans (Volkow et al., 2011; Jasinska et al., 2014; Uhl et al., 2019). One might speculate that modulation of VTA activity by NA contributes to counteracting or normalizing these patterns of reactivity – a hypothesis which will require further exploration and direct verification. In addition to changes in the DA system, NAergic signaling could also undergo allostatic plasticity in drug-experienced individuals, such as loss of autoreceptor functionality of α_2_-ARs normally regulating NA release (Doucet et al., 2013; Fox et al., 2015; Schmidt et al., 2019). Together, this could lead to altered NA release in response to optogenetic stimulation, thus explaining why manipulating LC terminals affects drug, but not food seeking. Notably, given that alcohol seeking is also susceptible to manipulation of LC output (Deal et al., 2020), it is likely that the phenomenon extends beyond psychostimulants and is common for multiple drugs of abuse.

A recent study has demonstrated the efficacy of optogenetic LC stimulation in manipulating operant drug taking as well as drug seeking in extinction sessions (Deal et al., 2020). In that study, two patterns of LC stimulation – low frequency (tonic, 5 Hz) and high frequency (phasic, 50 Hz), non-contingently delivered during an operant session, produced distinctly opposite effects. Tonic stimulation enhanced alcohol consumption, while phasic stimulation resulted in a decrease in both consumption and alcohol seeking, i.e., lever pressing during extinction trials. Of note is the fact that these authors demonstrated that 50 Hz stimulation is capable of effectively inducing NA efflux in basolateral amygdala; moreover, in a previous study, they had shown that LC stimulation results in stimulation frequency-dependent NA release patterns in the prefrontal cortex that are similar to those of DA (Deal et al., 2019). This phasic stimulation pattern was effective at reducing alcohol intake and seeking, which the authors tentatively explained as potential distraction or interference with attentional processing during an operant session. Here, we extend these findings by showing that stimulation of the LC-VTA circuit is sufficient to alter drug seeking. Moreover, the temporal window in which NA activity is manipulated during drug seeking behavior determines the outcome.

How, then, are the opposite effects on CS-induced drug seeking realized by contingent versus non-contingent optogenetic manipulation? Phasic activity of LC neurons has been shown to regulate momentary shifts in attention and performance, essentially focusing perceptive and cognitive resources towards motivationally relevant (salient) stimuli, a hypothesis dubbed “adaptive gain theory” (Aston-Jones and Cohen, 2005). More recently, this concept has been expanded by Mather et al. to include putative mechanisms in the form of cross-talk between NA and glutamate at active release sites (“hotspots”); this latter account has been dubbed GANE, “glutamate amplifies noradrenergic effects” (Mather et al., 2016; Poe et al., 2020). It proposes that a reciprocal feedback loop involving local glutamatergic activity and NA release, mediated *via* receptors located on NAergic and glutamatergic terminals as well as local astrocytes, supports a “winner-takes-more,” input-specific potentiation of salient stimuli processing.

Inspired by this theoretical framework, we speculate that NA acts in the VTA in concert with glutamatergic inputs onto DA neurons and GABAergic interneurons to shape adaptive gain, by facilitating highly-active excitatory inputs (Velásquez-Martinez et al., 2012; Williams et al., 2014), perhaps in combination with intrinsic inhibition *via* direct or indirect actions on DA neurons (Paladini and Williams, 2004; Pradel et al., 2018), similarly to how lateral inhibition would work in sensory systems. During cocaine self-administration, the animal is presented with at least two distinct contingencies, and thus – two successive windows of opportunity for behavioral response and associative learning to take place. The first contingency links the experimental context to the availability of CS and the opportunity to engage in previously reinforced operant behavior (i.e., the presence of levers), while the second contingency ensues immediately after a lever press, when the CS-US relation is processed (Torregrossa et al., 2011). These events then work in concert to drive drug seeking (Taylor et al., 2009; Torregrossa et al., 2011; Torregrossa and Taylor, 2016), analogous to how in SUD patients, responses to drug cues in terms of brain activity as well as self-reported craving also vary by context, e.g., expected drug availability (Crombag et al., 2008; Jasinska et al., 2014). When an animal is undergoing a session of cocaine seeking under extinction conditions, the two contingencies are present, but one of them has now changed: the levers and corresponding CS are available, but the US (cocaine) is not.

We hypothesize that in an intact animal, during a session of cocaine seeking, NA is tonically elevated in response to the “occasion setter” context, in anticipation of previously acquired cues and in preparation for the availability of operant behavior. Subsequently, LC phasically fires in response to the “CS – no US” mismatch, facilitating extinction – in agreement with the finding that a shift in reward contingency between stimuli results in robust phasic LC activity and NA release (Sara and Segal, 1991). Thus, while the animal is in an occasion-setting context, elevated NA tone acts in a straightforward manner to promote arousal and motivation to engage in goal-directed behavior (Luján et al., 2019), since there is no incongruity between the expected and obtained result (i.e., the animal is in a context where operant behavior is available and results in CS presentation). Conversely, immediately after the lever is pressed, there is a mismatch between the expected result (CS-US pairing) and the actual result (no US).

Here, we posit that these two time windows were separately targeted by optogenetic inhibition or stimulation. Non-contingent optogenetic inhibition presumably suppressed NA release in VTA for ∼30% of the total time spent by the animal in the experimental context. This – analogous to pharmacological blockade of α_1_-ARs – attenuated drug seeking by decreasing context-dependent anticipation and arousal, resulting in less drive to pursue operant responding based on the familiar occasion-setting context. The opposite effect has been shown previously when NA signaling was enhanced by intra-VTA α_1_-AR agonist or α_2_-AR blockade (Solecki et al., 2018). Conversely, contingent activation of opsins expressed on the noradrenergic afferent in the VTA, leading presumably to inhibition or stimulation of NA release upon lever press, respectively, either attenuated, or facilitated the phasic LC signal in response to CS-US mismatch, resulting in dampened or enhanced behavioral flexibility – manifesting here as extinction learning.

In summary, we report a time-dependent modulation of VTA circuitry – and, consequently, behavior – by NAergic LC afferents. Given that adrenergic drugs have been used for SUD treatment, a better understanding of the time- and context-dependent mechanisms involved could be useful e.g., for combining pharmacological intervention with extinction therapy aimed at targeting occasion setters versus discrete cues in patients (Taylor et al., 2009; Jobes et al., 2011; Newton et al., 2012; Shorter et al., 2013; Fox and Sinha, 2014). An important caveat for such potential applications is that male rats were used in the present study. Sex differences in the noradrenergic and corticotropin release factor systems have been described in rats (e.g., Cason et al., 2016; den Hartog et al., 2020), and there is significant clinical evidence for sex- and gender-related variability in PSTD and SUD (Koob, 2021; Upadhyay et al., 2021). Further work will also be needed to identify the inputs and mechanisms involved on both time-scales. In particular confirming, with electrophysiological, pharmacological and imaging tools, whether the VTA-LC circuitry, as opposed to off-target or antidromic excitation of broader LC connections, is sufficient for explaining the effects of optogenetic manipulation of VTA-projecting LC terminals (McCall et al., 2017), as well as whether the co-activation of glutamatergic and phasic NA release by afferents from LC (as per the GANE hypothesis) is involved in the observed outcomes. Additional complexity can also be expected in more complicated tasks, and in other experimental systems, such as in primates, where e.g., orientation and reaction to visual cues, also modulated by NA, affect task performance (Jahn et al., 2018; Totah et al., 2019).

## Data availability statement

The raw data supporting the conclusions of this article will be made available by the authors, without undue reservation.

## Ethics statement

The animal study was reviewed and approved by 2nd Local Institutional Animal Care and Use Committee Institute of Pharmacology Polish Academy of Sciences in Kraków, Poland.

## Author contributions

WS designed the study, wrote the manuscript, performed surgeries, and performed and analyzed behavioral experiments. MK performed surgeries and contributed extensively to writing the manuscript. MW and KZ performed behavioral experiments. MW, KZ, and KK contributed to histological verification. KK performed immunohistochemical stainings and confocal microscopy. ZR and MK provided expertise in the confocal microscopy. RP provided critical revision of the manuscript. All authors reviewed content and approved final version for publication. All authors contributed to the article and approved the submitted version.
