# Supplementary material for: Regulation of cocaine seeking behavior by locus coeruleus noradrenergic activity in the ventral tegmental area is time- and contingency-dependent
Source: Front Neurosci. 2022 Aug 5;16:967969. doi: 10.3389/fnins.2022.967969 (PMC9388848; doi:10.3389/fnins.2022.967969)
Supplement: Supplementary file 1 [file Data_Sheet_1.docx]

Regulation of cocaine seeking behavior by locus coeruleus noradrenergic activity in the ventral tegmental area is time- and contigency-dependent

**Supplementary Methods and Materials**

**Subjects**

Animals (250-280 g) were housed five per cage in a temperature and humidity-controlled room (20-22 ºC, 40-50% humidity), on a 12 hour light/dark cycle (lights on at 7 am), with *ad libitum* access to food and water, unless specified otherwise. Care was taken to minimize experimental animal use, with cohort sizes chosen based on the assumption of effect size and success ratios in line with our previous studies (Solecki *et al*, 2018).

Rats were anesthetized with ketamine HCl (100 mg/kg, i.m., Biowet-Puławy, Poland) and xylazine (10 mg/kg, i.m., Biowet-Puławy, Poland) and placed in a stereotaxic frame (Stoelting Europe, Ireland) for viral microinjections. First, a small sagittal skin incision at the top of the scalp and a small hole in the skull above the LC were made. Next, AAVs (virus titer: 4.4 × 10^12^ virus molecules/ml) were administered into the VTA (0.1 µl/1 min; total volume of 0.5 µl). Stereotaxic coordinates used for the LC microinjection were: AP -9.8 mm, ML ±1.5 mm, DV -7.0 mm from the Bregma. Finally, either the incision above the skull was sutured with silk sutures or the rats underwent intra-VTA optic-fiber implantation procedure as described below. Rats were given at least 4 weeks to recover after the procedure to ensure effective virus transduction (Solecki et al., 2019).

**Post-surgery care**

All animals were given an anti-inflammatory and analgesic drug (Tolfedine 4%, i.p., Vetoquinol Biowet, Poland) and glucose (5 ml) to prevent dehydration during post-surgery recovery. For the first three days after the operations, animals were treated with antibiotics added to the drinking water (Sul-Tridin 24%, Biowet-Pulawy, Poland).

**Immunohistochemistry**

Animals were anesthetized with pentobarbital (150 mg/kg i.p., Biowet-Pulawy, Poland), and perfused transcardially with 0,9 % NaCl followed by 4% formalin (Chempur, Poland). Immediately after perfusion, animals were decapitated; their brains were removed and post-fixed in PBS containing 4% formalin at 4 °C. Next, brains were sliced with the vibratome (model VT1200, Leica Biosystems, Germany) to coronal brain sections (50 μm). For each animal 6–12 sections were acquired at the level of the VTA. Slices were placed in 0,1% natrium azide solution (K305.1; Roth, Germany) for immunohistological procedure. For the tyrosine hydroxylase (TH) and enhanced yellow fluorescent protein (EYFP) staining, sections were immersed in a blocking solution consisting of 5% normal goat serum (G9023; Sigma Aldrich, Germany) dissolved in PBST (0,5% Triton X-100 with 0,1% PBS). Cell nuclei were identified using the 4′,6-Diamidino-2-phenylindole dihydrochloride (1µg/1ml, D9542, Sigma Aldrich, Germany).

**Evaluation of the efficiency and specificity of virus transduction**

The efficiency and specificity of the transfections were evaluated by confocal microscopy and ImageJ software by comparing the EYFP cells with TH immunoreactive cells. First, images of the coronal brain slices (AP: 4.6-5.6 mm from the Bregma) were taken with the confocal microscopy. The analyzed images had a resolution of 3300x3300 pixels (1416.99 x 1416.99 µm). Two ImageJ macros downloaded from the <https://imagej.nih.gov/ij/macros/tools/>  were used. Macro SomaRoiTool.txt was used to create a circular ROI (region of interest) on mouse click. The area of ROI was equal 80 pixels (14.75 µm^2^). Next, the background grey levels both in the red (TH staining) and green (EYFP) channels were measured. Then, the ROIs were placed on the cell silhouettes in the red channel and the mean grey level was measured in green and red channels simultaneously, with macro MeasureStack.txt. The cell was regarded as efficiently transfected if the intensity of fluorescence in the ROI in the green channel was at least twice higher than the background. Similarly, in order to evaluate the transfection specificity, the ROIs were placed on cell silhouettes in the green channel. Intensity of staining at least twice higher than background in the red channel was accepted as the criterion of specificity.

**Cocaine self-administration**

SD-TH-Cre+ rats were anesthetized with ketamine HCl (100 mg/kg, i.p., Sigma-Aldrich) and xylazine (10 mg/kg, i.p., Sigma-Aldrich) and implanted with a silastic catheter in the external jugular vein, as described previously (Solecki et al., 2018). Cocaine self-administration training was performed as previously described (Solecki et al., 2018) and started after 9 days of recovery from surgery and was preceded by 2 days of food restriction to ~ 90% of free-feeding levels and 2 days (3 × 1 h training sessions daily, with 1 h between each session) of food self-administration training. For food self-administration, one day prior to training, 20–30 food pellets (45 mg; BioServ, NJ, USA) were placed into the home cage to introduce the rats to the new food. Next, rats were trained in standard operant chambers illuminated by a house light (Med Associates, St. Albans, USA) under a fixed ratio 1 (FR1) schedule of reinforcement during which lever press (only one lever was extended into the chamber) led to food pellet delivery. All rats acquired operant responding for food (> 20 lever presses). Subsequently, all rats began cocaine self-administration training with the FR1 schedule of reinforcement during which each active lever press (two levers were extended) led to an intravenous cocaine infusion (0.18 mg over 6 s, ~0.5 mg/kg) and the conditional stimulus (CS) presentation (tone + stimulus light for 6 s) in standard operant chambers illuminated by a house light. Each active lever press was followed by a 20 s timeout during which lever pressing had no programmed consequences. Similarly, inactive lever presses had no programmed consequences. Each rat received 2-h daily training sessions for 9-10 consecutive days. The acquisition of stable self-administration behavior was defined as less than 15% variability in total active lever presses over 3 consecutive days. Animals that did not acquire stable self-administration behavior (10 or more active lever presses during 3 consecutive sessions) were excluded from the study (n=6).

**Conditioned place aversion**

All rats were habituated to handling by the experimenters for at least five consecutive days prior to behavioral testing. All rats were transferred into the testing room 24 h before testing, allowing acclimatization to the room for at least 4 h. In the room, rats were weighed and handled.

The conditioned place aversion apparatus consisted of two conditioning chambers separated by a central platform. One of the chambers had white Plexiglas walls with vertical black stripes and a black Plexiglas floor with small holes in it. The second chamber had white Plexiglas walls with horizontal black stripes and a rough black floor. The distinct colors and textural floor cues served as conditioned stimuli. The central platform had gray and white Plexiglas walls and a smooth grey floor. The conditioning chambers could be separated from the central platform by sliding walls. Both chambers and the central platform were dimly illuminated with white light (~10 lux in the middle of each chamber). Each rat was semi-randomly assigned to a specific laser photo-inhibition protocol (1 s every 9 s or 6 s every 12 s). The apparatus was kept free of urine and feces, and the floors were washed and dried after each rat.

**Data Analysis**

Behavioral effects of photo-modulation in the RT-CPA and CPA were analyzed using Student's t test, one-way ANOVA or a two-way repeated-measures ANOVA (GraphPad Software, San Diego, CA, USA, Statistica 12.5, Stat-Soft, Poland). The effects of intra-VTA photo-modulation on the lever presses during the CS-induced cocaine- or food seeking tests were analyzed using two-way ANOVA. For analysis of lever responding during cocaine or food self-administration, a three-way repeated-measures MANOVA was performed. For analysis of number of cocaine infusions or food pellets earned during self-administration, a two-way repeated-measures ANOVA was performed. If there was a significant main effect or a significant interaction, a subsequent Newman–Keuls *post hoc* analysis was performed. Table S2 presents the factors and levels of ANOVA according to the photo-modulation and performed behavioral tests. Assumptions for ANOVA were assessed based on visual inspection of diagnostic and per-group plots (not shown). Statistical significance was set at P < 0.05. All results values are presented as the means + SEM.
