# Supplementary material for: Regulation of cocaine seeking behavior by locus coeruleus noradrenergic activity in the ventral tegmental area is time- and contingency-dependent
Source: Front Neurosci. 2022 Aug 5;16:967969. doi: 10.3389/fnins.2022.967969 (PMC9388848; doi:10.3389/fnins.2022.967969)
Supplement: Supplementary file 2 [file Table_1.doc]

Table S1. Number of subjects with the VTA optic-fibers misplacements. EYFP – rats with AAV-DIO-EYFP micro-infusion into the LC, Arch3.0 – rats with AAV-DIO-Arch3.0-EYFP micro-infusion into the LC, n.a. – not applicable. The total number of subjects in each treatment group is indicated in parentheses.

| Behavioral test | AAVs treatment | VTA |
| --- | --- | --- |
| Cocaine seeking with noncontingent photo-inhibition | EYFP | n.a. (8) |
|  | Arch3.0 | 3 (11) |
| Cocaine seeking with contingent photo-inhibition | EYFP | n.a. (8) |
|  | Arch3.0 | 1 (6) |
| Cocaine seeking with contingent photo-stimulaton | EYFP | 1 (8) |
|  | ChR2 | 1 (10) |
| Food seeking with contingent photo-stimulaton | EYFP | n.a (8) |
|  | ChR2 | 3 (9) |
