# Supplementary material for: Regulation of cocaine seeking behavior by locus coeruleus noradrenergic activity in the ventral tegmental area is time- and contingency-dependent
Source: Front Neurosci. 2022 Aug 5;16:967969. doi: 10.3389/fnins.2022.967969 (PMC9388848; doi:10.3389/fnins.2022.967969)
Supplement: Supplementary file 3 [file Table_2.docx]

Table S2. A table listing the factors and levels of ANOVA according to the AAVs micro-infusions and behavioral tests.

| **Treatment** | **Test** | **Statistics** | **Factors** | **Levels** |
| --- | --- | --- | --- | --- |
| AAV-Arch3.0 into the LC | CS-induced cocaine seeking during WD 3 – noncontingent photo-inhibition | Two-way ANOVA with Newman Keuls *post hoc* | Treatment | Control (EYFP); 6 s photo-inhibition |
|  |  |  | Lever | Active, inactive |
|  |  |  | Interaction | Treatment × lever |
| AAV-Arch3.0 into the LC | CS-induced cocaine seeking during WD 3– contingent photo-inhibition | Two-way ANOVA with Newman Keuls *post hoc* | Treatment | Control (EYFP); 6 s photo-inhibition |
|  |  |  | Lever | Active, inactive |
|  |  |  | Interaction | Treatment × lever |
| AAV-ChR2 into the LC | CS-induced cocaine seeking during WD 3– contingent photo-stimulation | Two-way ANOVA with Newman Keuls *post hoc* | Treatment | Control (EYFP); 60 Hz photo-stimulation |
|  |  |  | Lever | Active, inactive |
|  |  |  | Interaction | Treatment × lever |
| AAV-ChR2 into the LC | CS-induced food seeking during WD 3– contingent photo-stimulation | Two-way ANOVA with Newman Keuls *post hoc* | Treatment | Control (EYFP); 60 Hz photo-stimulation |
|  |  |  | Lever | Active, inactive |
|  |  |  | Interaction | Treatment × lever |
| AAV-Arch3.0 into the LC | Open field test in cocaine-abstinent rats – distance over time | Repeated-measures two-way ANOVA with Newman Keuls *post hoc* | Treatment | Control (EYFP); 6 s photo-inhibition |
|  |  |  | Time | 5, 10 and 15 min |
|  |  |  | Interaction | Treatment × time |
| AAV-ChR2 into the LC | Open field test in cocaine-abstinent rats – distance over time | Repeated-measures two-way ANOVA with Newman Keuls *post hoc* | Treatment | Control (EYFP); 60 Hz photo-stimulation |
|  |  |  | Time | 5, 10 and 15 min |
|  |  |  | Interaction | Treatment × time |
| AAV-ChR2 into the LC | Open field test in cocaine-naive rats – distance over time | Repeated-measures two-way ANOVA with Newman Keuls *post hoc* | Treatment | Control (EYFP); 60 Hz photo-stimulation |
|  |  |  | Time | 5, 10 and 15 min |
|  |  |  | Interaction | Treatment × time |
| AAV-Arch3.0 into the LC | RT-CPP in cocaine-abstinent rats – % time in laser-paired chamber | Repeated-measures two-way ANOVA with Newman Keuls *post hoc* | Treatment | Control (EYFP); 6 s photo-inhibition |
|  |  |  | RT-CPP phase | Preconditioning, conditioning 1, conditioning 2, postconditioning |
|  |  |  | Interaction | Treatment × RT-CPP phase |
| AAV-ChR2 into the LC | RT-CPP in cocaine-abstinent rats – % time in laser-paired chamber | Repeated-measures two-way ANOVA with Newman Keuls *post hoc* | Treatment | Control (EYFP); 60 Hz photo-stimulation |
|  |  |  | RT-CPP phase | Preconditioning, conditioning 1, conditioning 2, postconditioning |
|  |  |  | Interaction | Treatment × RT-CPP phase |
| AAV-ChR2 into the LC | RT-CPP in cocaine-naive rats – % time in laser-paired chamber | Repeated-measures two-way ANOVA with Newman Keuls *post hoc* | Treatment | Control (EYFP); 60 Hz photo-stimulation |
|  |  |  | RT-CPP phase | Preconditioning, conditioning 1, conditioning 2, postconditioning |
|  |  |  | Interaction | Treatment × RT-CPP phase |
| AAV-Arch3.0 into the LC | RT-CPP in cocaine-abstinent rats – time in chambers during preconditioning | Two-way ANOVA with Newman Keuls *post hoc* | Treatment | Control (EYFP); 6 s photo-inhibition |
|  |  |  | Chamber | Laser-paired, center, control |
|  |  |  | Interaction | Treatment × chamber |
| AAV-ChR2 into the LC | RT-CPP in cocaine-abstinent rats – % time in laser-paired chamber | Repeated-measures two-way ANOVA with Newman Keuls *post hoc* | Treatment | Control (EYFP); 60 Hz photo-stimulation |
|  |  |  | Chamber | Laser-paired, center, control |
|  |  |  | Interaction | Treatment × chamber |
| AAV-ChR2 into the LC | RT-CPP in cocaine-naive rats – % time in laser-paired chamber | Repeated-measures two-way ANOVA with Newman Keuls *post hoc* | Treatment | Control (EYFP); 60 Hz photo-stimulation |
|  |  |  | Chamber | Laser-paired, center, control |
|  |  |  | Interaction | Treatment × chamber |
