# Supplementary figures and images for: Regulation of cocaine seeking behavior by locus coeruleus noradrenergic activity in the ventral tegmental area is time- and contingency-dependent
Source: Front Neurosci. 2022 Aug 5;16:967969. doi: 10.3389/fnins.2022.967969 (PMC9388848; doi:10.3389/fnins.2022.967969)

**A**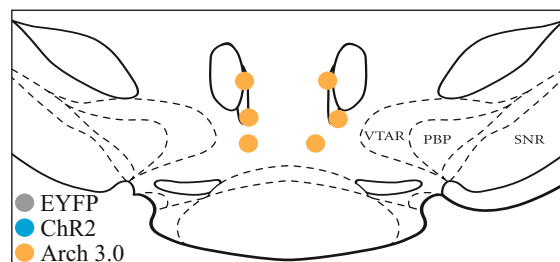 $-4.8 \pm 0.2$  mm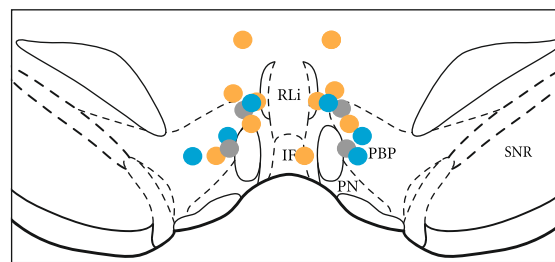 $-5.2 \pm 0.2$  mm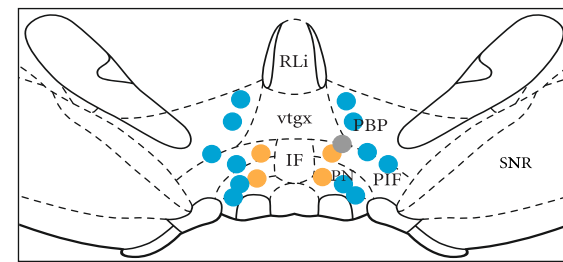 $-5.5 \pm 0.2$  mm**B**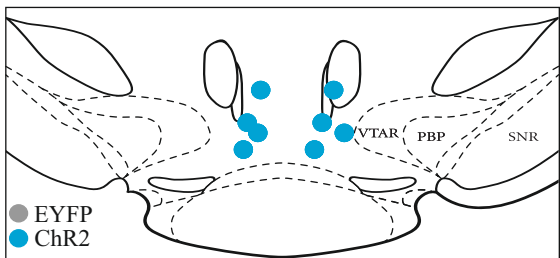 $-4.8 \pm 0.2$  mm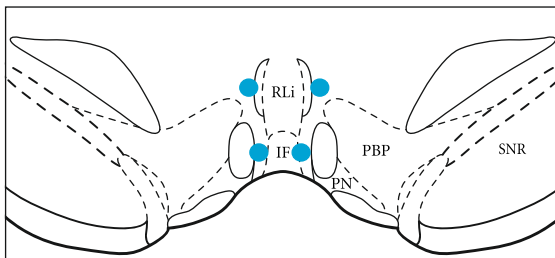 $-5.2 \pm 0.2$  mm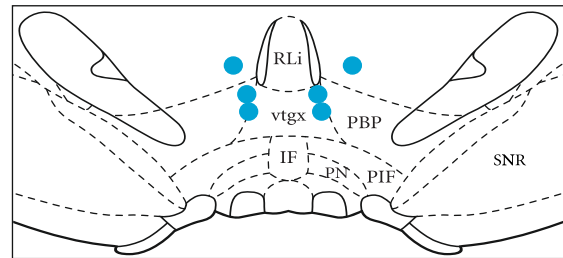 $-5.5 \pm 0.2$  mm**C**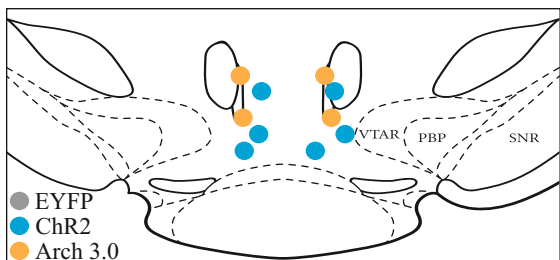 $-4.8 \pm 0.2$  mm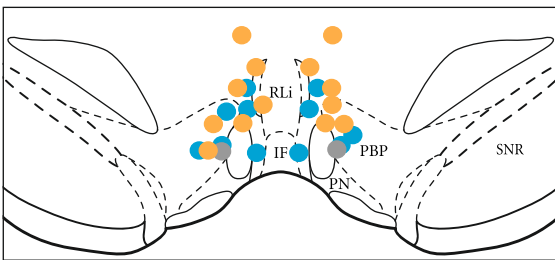 $-5.2 \pm 0.2$  mm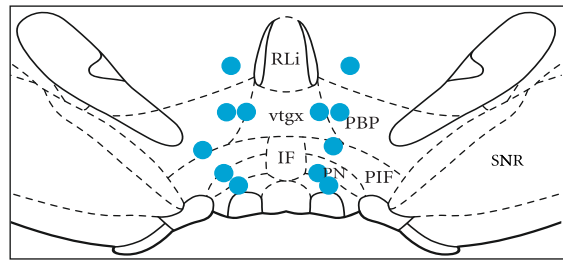 $-5.5 \pm 0.2$  mm**D**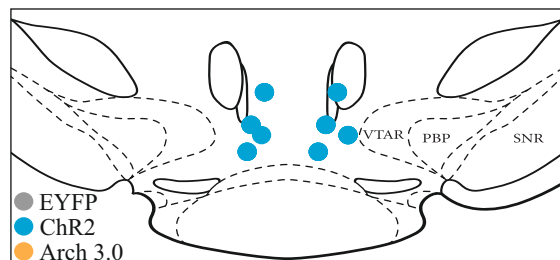 $-4.8 \pm 0.2$  mm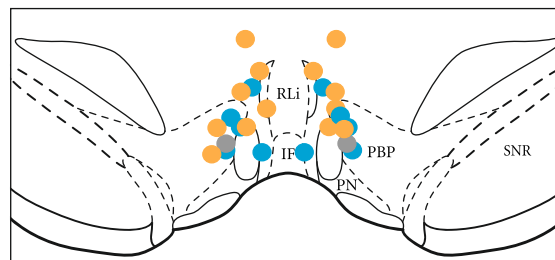 $-5.2 \pm 0.2$  mm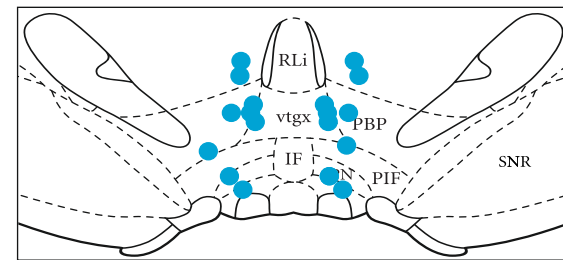 $-5.5 \pm 0.2$  mm

Supplement: Supplementary file 4 [file Image_1.pdf]

**A**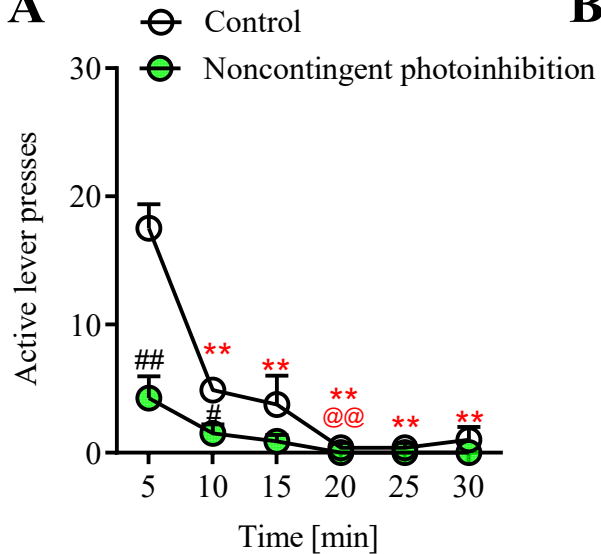**B**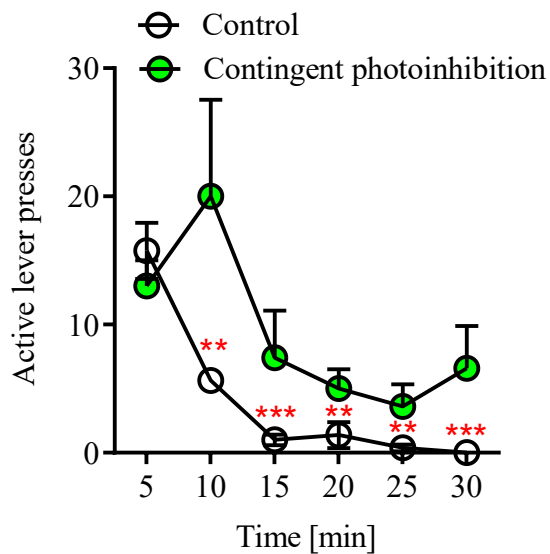**C**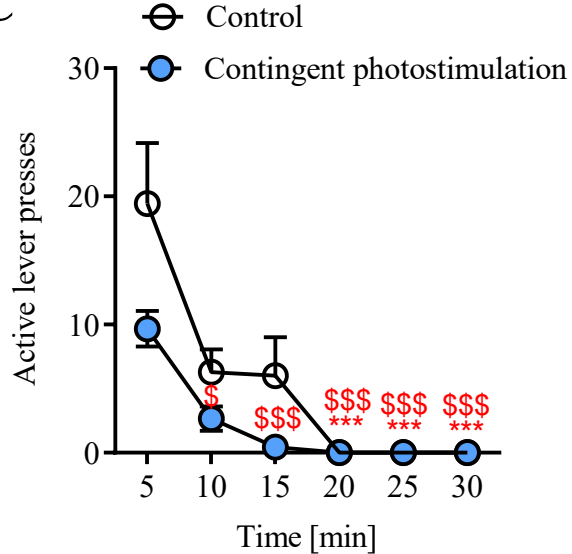

Supplement: Supplementary file 5 [file Image_2.pdf]
